# Editorial: Emotional Intelligence and Cognitive Abilities

**DOI:** 10.3389/fpsyg.2016.00955

**Published:** 2016-06-23

**Authors:** Pablo Fernández-Berrocal, Purificación Checa

**Affiliations:** ^1^Department of Basic Psychology, Faculty of Psychology, University of MálagaMálaga, Spain; ^2^Department of Psychology, Faculty of Education Science, University of CádizCádiz, Spain

**Keywords:** emotions, emotional intelligence, cognitive abilities, personality, health

A quarter-century after Peter Salovey and John Mayer introduced the concept of emotional intelligence (EI; Salovey and Mayer, 1990), it remains an important and growing research area. EI is usually analyzed from the perspective of the ability model or mixed models. The ability model focuses on an individual's mental abilities to apply information provided by emotions for the improvement of cognitive processing. Mixed models conceptualize EI as the combination of mental abilities, stable behavioral traits and personality variables (see Mayer et al., 2008). The present research topic brings together 14 articles that, building on previous studies (Mayer et al., 2008; Fernández-Berrocal et al., 2014; Webb et al., 2014), analyze the relevance of EI in different domains of daily life, including health-related outcomes, social functioning, and academic and workplace performance. These articles approach the problem from different theoretical perspectives using different measurements.

The research topic opens with a review by Peña-Sarrionandia et al. on studies that apply the emotion regulation conceptual framework to understanding how emotion regulation processes underlie EI. This review offers an integrated view of the field that may help the reader better understand the other articles in this research topic.

Researchers continue to debate whether EI contributes significantly to emotional, cognitive, and social processes beyond the well-known contribution of IQ and personality traits. The link between cognitive ability and general intelligence is well-established, and evidence also suggests a link between cognitive ability and EI. Checa and Fernández-Berrocal show that the Managing Emotions dimension of EI measured by the Mayer-Salovey-Caruso Emotional Intelligence Test (MSCEIT) is negatively related to impulsivity during cognitive tasks. Boyatzis et al. report that the association of general intelligence with the behavioral dimension of EI differs from its association with other EI dimensions. Individuals with greater EI ability (based on the MSCEIT) perceive more available social support, after controlling for fluid intelligence and personality traits (Di Fabio). EI abilities have also been implicated in divergent thinking and motivational state (Takeuchi et al.). At the same time, not all studies suggest a significant contribution of EI beyond IQ or personality. Furnham et al. examine associations of personality traits with self- and other-reported EI (based on the Bar-on EQ-I) in business settings, and they find poor incremental benefit of this EI measure beyond personality testing. Together, these studies suggest that EI is a relevant and useful variable for understanding and explaining various human processes, though further research should clarify its importance relative to IQ and personality.

The importance of EI in cognitive and behavioral processes prompts the question: what is the brain system that supports EI? Studies have implicated the ventromedial and dorsolateral prefrontal cortex (Krueger et al., 2009), as well as the dopaminergic system (Takeuchi et al.).

Recent studies suggest that EI contributes to relationships among emotions, health, and well-being. Some articles in this research topic focus on this contribution in different contexts. Fernández-Abascal and Martin-Diaz suggest that dimensions of self-report EI are better predictors of Mental Health than of Physical Health in adults. Limonero et al. suggest that the Emotional Facilitation and Emotional Understanding branches of MSCEIT are related to previous mood states and to mood recovery after induction of negative mood. Elipe et al. analyze perceived EI as a moderator between cyber-victimization and its emotional impact on university students, while Amdurer et al. provides longitudinal data suggesting that EI predicts career satisfaction and success. Sánchez-Álvarez et al. confirm in a 2-year longitudinal study that positive and negative affect mediate the relationships between EI and life satisfaction in adolescents.

EI also appears to play important roles during early development (Mayer et al., 2008). This provides a clear example of where knowledge about EI may lead to useful interventions to improve quality of life. During middle childhood, reading and discussing children's books with emotional content can improve emotional competence (Kumschick et al.). Efforts to evaluate EI in different populations, such as deaf people (Mestre et al.), may open up promising avenues for designing interventions to improve integration, reduce alienation and enrich quality of life. Indeed, the research topic closes with an article by Cabello and Fernandez-Berrocal that provides the first evidence that implicit beliefs about emotions and EI may influence emotional abilities (based on MSCEIT). This may have important consequences for personal and professional EI training.

While this research topic answers some important questions from various perspectives, it does not address several other questions and it also raises new ones. For example, longitudinal and interventional studies should examine how different EI measures may interact with socio-demographic, educational and cultural factors, as well as with psychological factors such as IQ and personality, to impact personal, professional, and social outcomes (Cabello et al.; Cabello and Fernandez-Berrocal, 2015; Peña-Sarrionandia et al.).

We hope that this research topic highlights new knowledge and raises further questions about how individual differences in EI explain differences in cognitive processes and emotion regulation, which can have important real-world consequences.

## Author contributions

All authors listed, have made substantial, direct and intellectual contribution to the work, and approved it for publication.

## Funding

This research was financed by the Spanish Ministry of Economy (PSI2012-37490) and the Innovation and Development Agency of Andalusia, Spain (SEJ-07325).

### Conflict of interest statement

The authors declare that the research was conducted in the absence of any commercial or financial relationships that could be construed as a potential conflict of interest.
